# Canine Brucellosis: An Update

**DOI:** 10.3389/fvets.2021.594291

**Published:** 2021-03-02

**Authors:** Renato L. Santos, Tayse D. Souza, Juliana P. S. Mol, Camila Eckstein, Tatiane A. Paíxão

**Affiliations:** ^1^Departamento de Clínica e Cirurgia Veterinárias, Escola de Veterinária, Universidade Federal de Minas Gerais, Belo Horizonte, Brazil; ^2^Departamento de Patologia Geral, Instituto de Ciências Biológicas, Universidade Federal de Minas Gerais, Belo Horizonte, Brazil

**Keywords:** *Brucella canis*, brucellosis, dog, abortion, reproductive diseases, zoonosis

## Abstract

Canine brucellosis is an infectious and zoonotic disease caused by *Brucella canis*, which has been reported worldwide, and is a major public health concern due to close contact between dogs and humans. In dogs, canine brucellosis manifests with abortion outbreaks, reproductive failure, enlargement of lymph nodes, and occasionally affects the osteoarticular system, although the occurrence of asymptomatic infections in dogs are not uncommon. In humans, the disease is associated with a febrile syndrome, commonly with non-specific symptoms including splenomegaly, fatigue, and weakness. Infection of dogs occurs mostly by the oronasal route when in contact with contaminated tissues such as aborted fetuses, semen, urine, and vaginal secretions. In humans, contact with contaminated fluids from infected dogs is an important source of infection, and it is an occupational risk for veterinarians, breeders, laboratory workers, among other professionals who deal with infected animals or biological samples. The diagnosis in dogs is largely based on serologic methods. However, serologic diagnosis of canine brucellosis remains very challenging due to the low accuracy of available tests. Molecular diagnostic methods have been increasingly used in the past few years. Treatment of infected dogs is associated with a high frequency of relapse, and should be employed only in selected cases. Currently there are no commercially available vaccines for prevention of canine brucellosis. Therefore, development of novel and improved diagnostic methods as well as the development of efficacious and safe vaccination protocols are needed for an effective control of canine brucellosis and its associated zoonotic risk.

## Introduction

The term “brucellosis” refers to a disease that results from infection of humans and animals with *Brucella* spp. Although there are much more genetic variations among strains of *Escherichia coli* or serotypes of *Salmonella enterica* than among *Brucella* species (1), *Brucella* spp. are usually host restricted, which has been the traditional approach for naming the species. For instance, among classical *Brucella* spp., namely *B. melitensis, B. suis, B. abortus, B. canis, B. ovis*, and *B. neotomae* have small ruminants, pigs, cattle, dogs, sheep, and rodents as their preferred hosts, respectively (2). During the past recent years, the genus underwent a marked expansion with the recognition of additional species, including: *B. ceti* (3, 4), *B. pinnipedialis* (4), *B. microti* (5), *B. inopinata* (6), *B. papionis* (7), and *B. vulpis* (8), which have cetaceans (e.g., whales and dolphins), seals, common vole (*Microtus arvalis*), undetermined host, baboons, and wolves as preferential hosts, respectively.

Brucellosis is one of the most important zoonotic diseases worldwide (9, 10), and most of *Brucella* species are capable of infecting humans, although they have a highly variable zoonotic potential. *B. melitensis* is the most pathogenic species of *Brucella* for humans, with the exposure to only 1–10 CFU (colony forming units) being sufficient for establishment of infection, whereas *B. suis* and *B. abortus* have intermediate zoonotic potential. *B. canis* has the lowest zoonotic potential among the classic *Brucella* spp., and there are no documented cases of human infection with *B. ovis* (11, 12).

The pathobiology of brucellosis in livestock species have been extensively studied (13, 14), particularly due to its zoonotic and public health significance (11) as well as due to highly significant economic losses for the animal industry (15). In contrast, studies on canine brucellosis are mostly based on fragmented seroepidemiologic surveys (16). Importantly, canine infections with *B. canis* are widespread, which considering the limitations for accurate diagnosis in dogs and human patients (17), it certainly makes human brucellosis associated with *B. canis* a markedly neglected zoonotic disease. Therefore, the goal of this review was to provide an updated overview of the literature regarding different aspects *B. canis* infection in dogs as well as its relevance as a zoonotic disease, considering perspectives for improving the control of this disease.

## Epidemiology of Canine Brucellosis

*B. canis* is the most common cause of canine brucellosis (18, 19), although occasional infections with *B. melitensis, B. abortus*, or *B. suis* occur in dogs that have close contact with tissues or secretions of infected livestock animals, especially raw milk, aborted fetuses, and placentas (20, 21). Interestingly, *B. canis* was isolated from a lymph node of a cow, but the clinical and epidemiological implications of this finding is unknown (22).

In dogs, there is no evidence of breed predisposition, and the high number of well-documented outbreaks in beagles may be due to the broad use of this breed for research purposes (23–26). *B. canis* infection in dogs has been reported during outbreaks in kennels (23, 25–28) or serological surveys of stray and pet dogs (29–34). Serologic surveys demonstrated higher frequencies of *B. canis* infections in stray dogs when compared to responsibly owned dogs (30, 31), probably due to the absence of mating control in stray dogs, which favors transmission of the disease. In a recent study performed in Mississippi, the prevalence of *B. canis* infection in shelter dogs was 2.3%, but the prevalence in shelters varies from 0 to 8.6%, which indicates that a small number of shelters may have a high seroprevalence of brucellosis (34).

**Figure 1:**
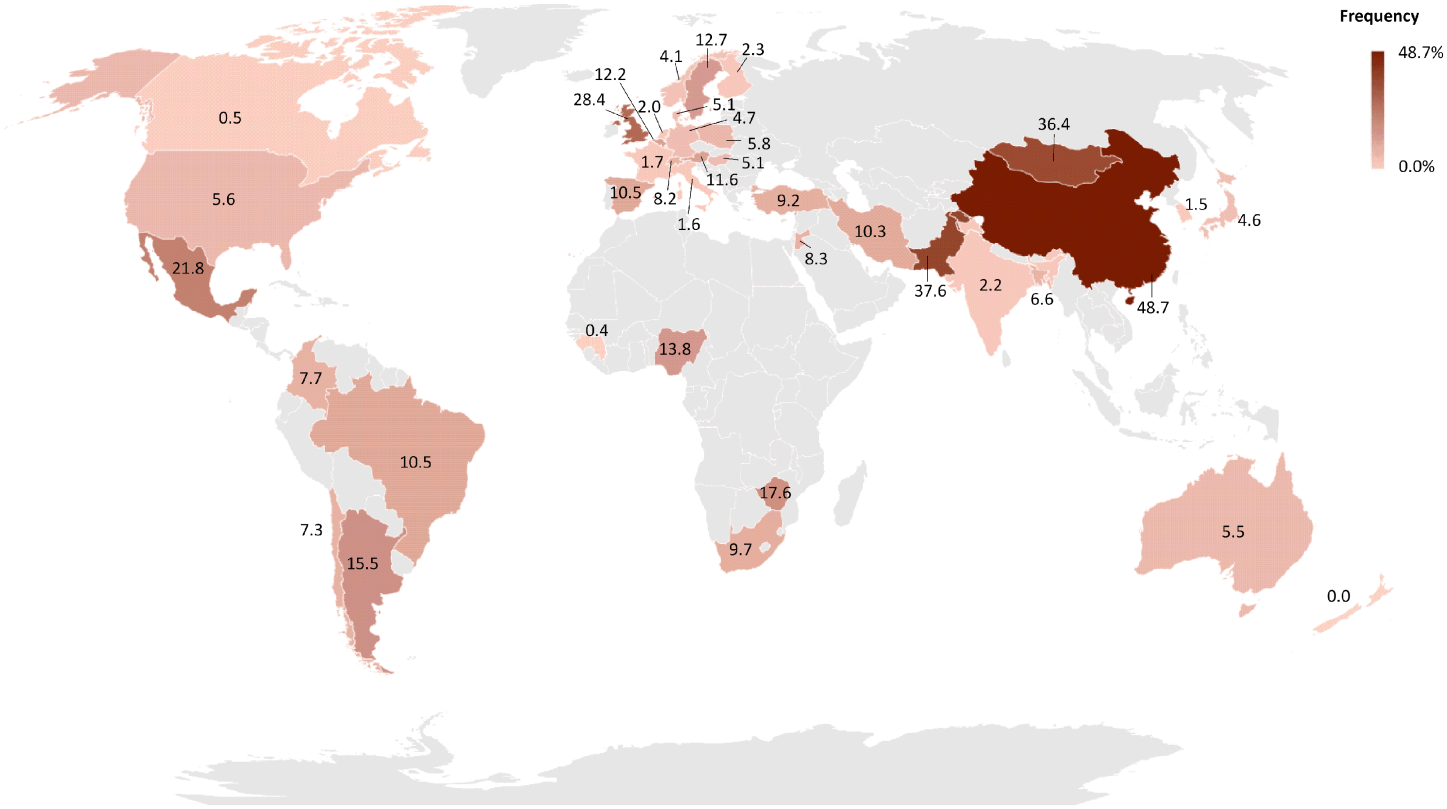
Worldwide distribution of the frequency of *B. canis* infected dogs by country. The frequency of each country was obtained by weighted average of the frequency reported in each study available on PubMed and Google Scholar. The map was generated using Microsoft Excel software. Regions in gray indicate countries without epidemiological surveys while countries showing patterns with black lines indicate countries with reports of dogs infected with *B. canis* but without studies of disease frequency.

*B. canis* was first isolated in 1966 from aborted fetuses in a Beagle kennel in the USA during an outbreak of abortions and reproductive failures (35). Since then, canine brucellosis caused by *B. canis* has been diagnosed in several countries (16, 19, 24, 27, 36), with the exception of Antarctica (37). Although the literature supports the notion that *B. canis* infection has a worldwide distribution (38), there are no consistent epidemiological studies assessing the prevalence of canine brucellosis. The lack of specific and efficient commercial laboratory tests may contribute to neglect the importance of canine brucellosis in many countries (17, 19, 39). The frequency of canine brucellosis in different parts of the world is represented in Figure 1. All studies employed for drawing the map (Figure 1) are cited in Supplementary Table 1 (40–125).

Studies using molecular tools, such as variable-number tandem repeat analysis (VNTR) and multiple loci VNTR analysis (MLVA), fatty-acid profiles or cellular fatty acid profiling (CFAP) methyl ester analysis, have been performed to identify markers in *B. canis* isolates to trace the origin and spread of infection in dogs and humans (27, 118, 126–129), although it may not always be possible.

In addition to being found in dogs, anti-*B. canis* antibodies have also been detected in wild canids (130, 131), and domestic and wild felines (131–133), although felines are considered resistant to brucellosis (130). There is serological evidence of antibodies anti-*B. canis* in other captivity (131) or free-ranging (131, 134) wild carnivore species, but the epidemiological importance of these species in canine brucellosis is unknown. Experimental infection demonstrated that *B. canis* is capable of infecting non-human primates (135), although natural infections have not been diagnosed in those animals.

Routes of *B. canis* infection include oral, nasal, conjunctival, and genital mucosa. Venereal transmission is important and occurs when *B. canis* is shed in the semen of infected dogs, particularly during the first 8 weeks after infection, although dogs may continue to shed *B. canis* intermittently in the semen for years (19, 24, 136). Infection can also be transmitted from an infected bitch to a susceptible male through contact with vaginal discharge during mating. *B. canis* may also be eliminated in the urine of male and female dogs. Puppies may be infected by intrauterine vertical transmission or after birth by the oronasal route through contaminated milk, contact with placental membranes or vaginal discharge after abortion (19, 137, 138). *B. canis* infection is associated with high neonatal mortality rates (46). Infected puppies that survive may become important sources of infection as permanent carriers of *B. canis* (26).

Large numbers of infectious bacteria are shed into the environment after abortions or through vaginal or seminal secretions. Therefore, fomites play an important role in the transmission of infection. Infected kennels must adopt stringent disinfection procedures and segregate feeding utensils and other materials to prevent spreading of infection (137). Canine blood transfusions can be a source of infection considering that *B. canis* causes intermittent but persistent bacteremia (18, 24). Transmission via blood-sucking fleas and ticks has not been confirmed, although *B. canis* have been isolated from these parasites (139).

Introduction of new dogs into a kennel, either as acquisitions or for breeding purposes without testing for *B. canis* favors the spreading of the disease (27). Usually, dogs are not properly tested since a successful diagnosis is laborious and challenging because it requires a combination of more than one laboratorial test and repeated sampling of biological specimens (17, 26, 28, 140).

## Human Infections With *Brucella canis*

A recent study demonstrated that *B. canis* is stealthier than pathogenic smooth *Brucella* (141), which supports the notion that *B. canis* may be under-diagnosed in human patients. The incidence of human brucellosis is estimated as half a million new cases per year, and this is considered an underestimation. The prevalence is extremely variable among different countries, and it is directly associated with infection in domestic animals and control policies (36, 142).

Human infections with *B. canis* were first reported in 1968, affecting individuals who had contact with infected dogs (23, 143). Although human infections with *B. canis* have been described in several countries, the prevalence of the disease is unknown (24, 36, 140, 144–147). Human infection with *B. canis* is considered self-limiting and occasional. It has been estimated that only 1% of the diagnosed human brucellosis are due to *B. canis* infection (146, 148). However, the incidence of human *B. canis* infection is may be underestimated.

Human *B. canis* infections are acquired through the oronasal route by direct contact with infected dogs, particularly by the contact with contaminated aborted fetuses or secretions. Laboratorial manipulation of the agent without protection is another relevant source of infection. As detailed in Table 1, for most cases of human infections with *B. canis*, there is an identifiable previous contact with infected dogs or contaminated biological materials in the laboratory (23, 31, 138, 143, 144, 149–152). Lucero et al. (152) described an outbreak of human brucellosis affecting three families who purchase puppies from an infected bitch, demonstrating high risk of transmission from infected dogs to their contacts. Children can also be considered a risk group due to close proximity to pet dogs (144).

**Table 1:** Profile of human patients infected with *Brucella canis*.

**Category**	**Frequency (%)**	
Sex	Woman	35.7 (10/28)
	Man	64.3 (18/28)
History of disease		21.4 (6/28)
Infection source	Laboratorial	10.7 (3/28)
	contact with infected/suspected dog	78.5 (22/28)
Age (years)	Below to 10	14.3 (4/28)
	>11 e < 20	17.8 (5/28)
	>21 e <50	46.4 (13/28)
	Above to 50	17.8 (5/28)
	Non-described	3.6 (1/28)

Human brucellosis is considered an occupational disease, so veterinarians, pet store workers, kennels employees and owners, dog caregivers, dog trainers, and laboratory technicians are professionals with the higher risk of occupational exposure to infection (23, 140, 144, 152, 153). A study of 306 asymptomatic adults with occupational exposure risk demonstrated a seroprevalence of 3.6% for *B. canis* (140). An interesting epidemiological study made by Monroe et al. (144) demonstrated higher prevalence of anti-*B. canis* antibodies in veterinarians and patients with unknown origin fever. Human *B. canis* infection may result from contact with aerosols formed during routine laboratory practices or accidental laboratorial exposure (23, 138, 151, 154). Importantly, manipulation of most *Brucella* spp., including *B. canis*, should be performed under biosafety level 3 conditions (155).

**Figure 2:**
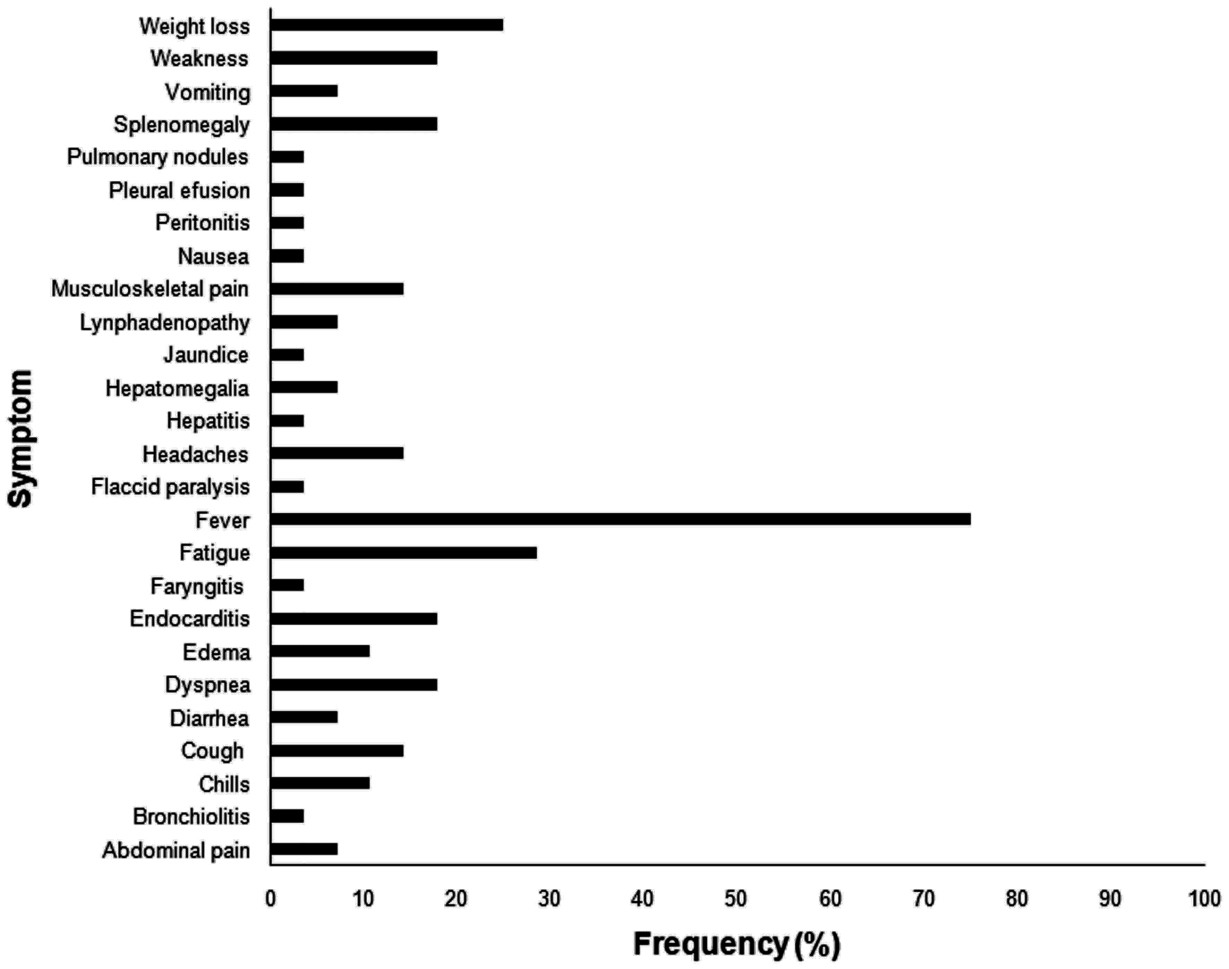
Frequency of symptoms reported in human brucellosis caused by *Brucella canis*. *All references used in the figure are listed in Supplementary Table 1.

Human brucellosis caused by *B. canis* resembles the clinical manifestations associated with other *Brucella* spp. infections (Figure 2), with unspecific symptoms, including intermittent fever, chills, sweating, loss of appetite, weight loss, fatigue, headaches, back pain or joint pain (146, 148, 156). Although *B. canis* is considered less pathogenic to human than other *Brucella* species, severe manifestations such as endocarditis, aneurysm, peritonitis, arthritis, osteomielitis, and epidural abscess have been described in *B. canis* infected patients (138, 149, 157–160). Neurobrucellosis, another important clinical manifestation of the disease in human patients, is usually due to *B. melitensis* infection, but there are reports of rare cases of neurologic disease associated with *B. canis* infection (161). Secondary neurological syndromes such as Guillain-Barré (138) have been associated with *B. canis* infection as well as with other *Brucella* spp. (162). Association of *B. canis* infection with other metabolic or immune diseases can aggravate brucellosis in human patients (138, 163–165). Indeed, immunodeficiency may be a risk factor for human infection with *B. canis* (163, 164).

Due to limitations of clinical or laboratorial diagnosis, human brucellosis due to *B. canis* is underdiagnosed, and its importance in public health is largely neglected (140, 166).

## Pathogenesis

Molecular mechanisms of pathogenesis are highly conserved among different species of *Brucella* spp. Therefore, unless stated otherwise, the mechanisms described here are common to the genus – not *B. canis*-specific. The goal of this section is not to provide a thorough literature review on *Brucella* spp. pathogenesis, which has already been reviewed (12, 167, 168), but to give an overview of *Brucella* pathogenesis with emphasis on the few studies that have focused specifically on *B. canis*.

The most common routes of *Brucella* infection are through the digestive or respiratory mucosa. Thus, a key step in *Brucella* pathogenesis is its ability to cross intact intestinal epithelia, particularly through M cells, in a completely stealthy fashion without activating innate immune response from the host (169). The two-component regulatory system BvrR/BvrS is required for *Brucella* spp. invasion and surveillance in phagocytic and non-phagocytic cells, specifically by recruiting GTPases, particularly Cdc42 (170). Lipopolysaccharide (LPS) is also considered an important virulence factor of *Brucella* spp. (171). Interestingly, naturally rough *Brucella* strains (due to the lack of O-polysaccharide chain of its LPS molecules) such as *B. canis* tend to invade host cells more efficiently than smooth strains, but they have lower survivability within host cells in culture or *in vivo* (172–174). Smooth LPS is protective against several host bactericidal mechanisms, including antimicrobial peptides, nitric oxide, and free radicals (171). Therefore, outer membrane proteins (Omp) also play a role in virulence (175, 176).

Earlier studies identified the *virB* operon-encoded *Brucella* type IV secretion system (T4SS) that is essential for intracellular survival and persistence *in vivo* (177, 178). This system translocates bacterial effector proteins directly into the host cell cytosol. In the absence of a functional T4SS, *Brucella* is not capable of directing the intracellular trafficking of the *Brucella*-containing vacuole toward the rough endoplasmic reticulum (RER), which constitutes the intracellular replicative niche for *Brucella* (179).

As mentioned above, most of the studies on *Brucella* pathogenesis do not involve *B. canis*, but a few particularities have been described. For instance, *B. canis* infection induces a poor pro-inflammatory response even in its preferential host, whereas this species is much less prone to induce inflammation than some of the smooth pathogenic *Brucella* species under experimental conditions, resulting in much lower induction of IFNγ production and inflammatory lesions (141).

## Pathology

In general, *Brucella* spp. infection in livestock results in reproductive disease, which is usually associated with abortion and placentitis in pregnant females, and epididymitis or orchitis in males (13, 14). In contrast, human brucellosis manifests as a febrile disease with a broader range of symptoms (11, 180). Here we will focus on gross and microscopic lesions that have been associated with *B. canis* infection in dogs.

**Figure 3:**
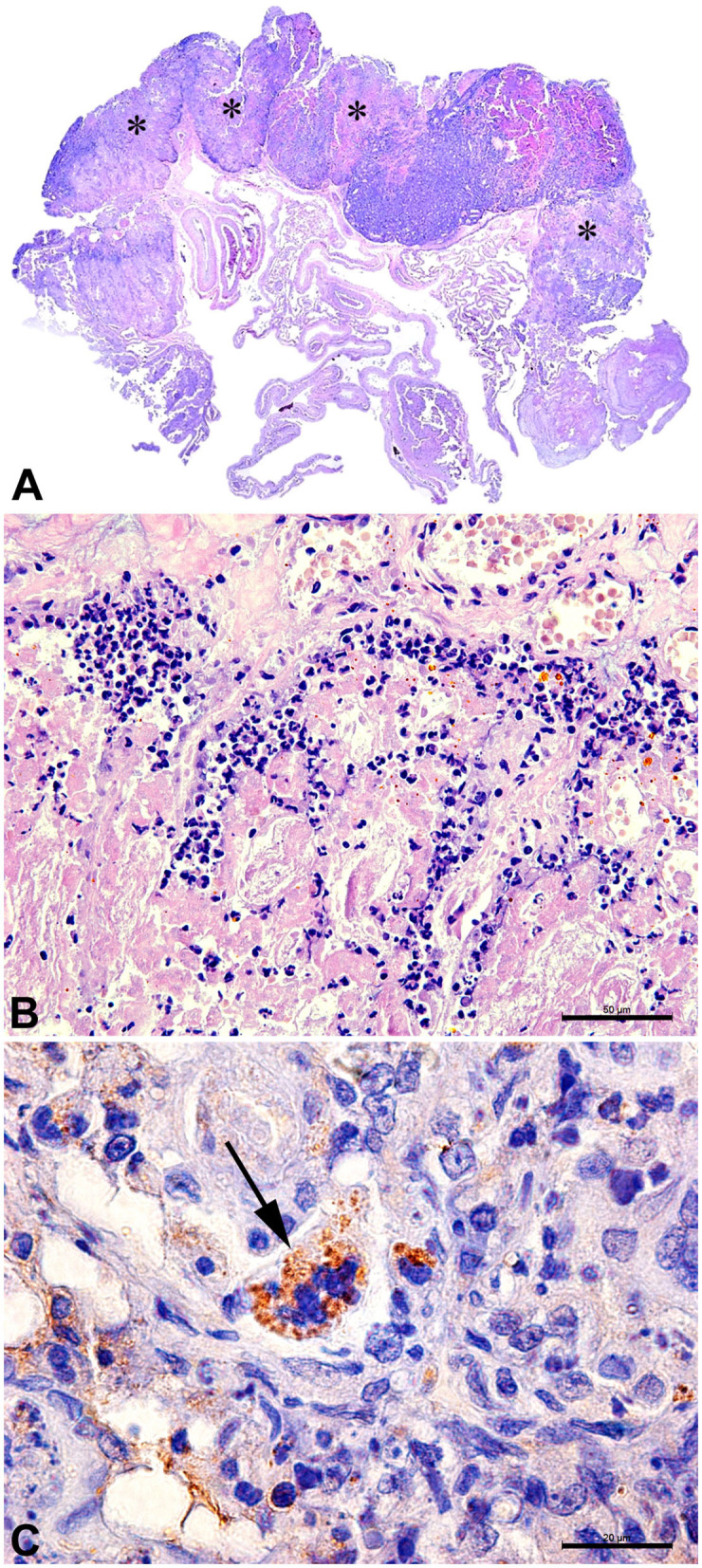
Canine placenta. **(A)** Sub-macroscopic view of the placenta with multiple focally extensive areas of necrosis (*) characterizing a necrotizing placentitis. **(B)** Necrotic tissue and marked neutrophilic inflammatory infiltrate. HE Bar = 50 μm. **(C)** Immunohistochemistry for detection of *Brucella* sp.: trophoblast with intracytoplasmatic immunolabed coccobacilli (arrow). Bar = 20 μm.

*B. canis* has been originally identified as a cause of abortion in dogs. In bitches, the infection is usually associated with metritis, placentitis, and abortion, with focal necrosis of the chorionic villi and numerous bacteria within trophoblastic cells (Figure 3) (35). Aborted fetuses may have bronchopneumonia, myocarditis, renal hemorrhage, lymphadenitis, and hepatitis (35). These *B. canis*-induced lesions in the canine pregnant uterus and fetuses are similar to lesions induced by *Brucella* spp. in other animal species (13, 14), although *B. canis* has been detected in a wide range of tissues from naturally infected neonates, including stomach, intestines, kidney, central nervous system, umbilicus, liver, lungs, lymph nodes, and spleen (46). In addition to abortion, *B. canis* infection is associated with the birth of weak puppies with a high neonatal mortality rate (35, 46). *B. canis*-infected male dogs develop epididymitis, orchitis, and prostatitis, which result in poor sperm quality and infertility (181). Epididymitis seems to be a more common primary lesion than orchitis (35), which contrasts with cattle that often develop orchitis due to *B. abortus* infection, but it is similar to *B. ovis* infection in rams, which causes primarily epididymitis (14). In addition to epididymitis, *B. canis* infection in male dogs is also often associated with inflammatory changes in the prostate gland and renal pelvis (182).

Less common manifestations of canine brucellosis include ocular and skeletal lesions, which are characterized by mild-to-moderate anterior uveitis, iris hyperpigmentation, vitreal inflammatory infiltrate, and multifocal chorioretinitis (183) and diskospondylitis (184, 185), respectively.

## Clinical Manifestation

Most *B. canis* infected dogs do not develop any clinical signs other than enlarged lymph nodes. Some of them may present loss of vigor and reproductive failure, and most of the abortions commonly between 45 and 55 days of gestation (35, 186), although in some cases they occur in the initial phase of gestation (between 10 and 35 days), when it is easily confused as failure in conception (19, 35).

Reproductive failure and interrupted whelping pattern have also been reported in association with *B. canis* infection (187). There may be repeated and consecutive abortions or alternated abortions and normal whelping, which affects infected females that are otherwise healthy. However, bitches are occasionally reported to be depressed for several weeks (35). Vaginal discharges are common after abortion, with variable duration (from 1 to 6 weeks), amount and appearance of the exudate, which is usually serosanguineous, but may be viscous and grayish green (16, 35).

Stillbirth or birth of weak puppies and neonatal death are also often associated with *B. canis* infection, but infected and apparently healthy puppies may be present in the same litter (26, 35, 46, 187). In some cases, enlarged lymph nodes are still observed in infected 2-month-old puppies (35), and bacteremia persists until at least 5 months of age (26). Surviving puppies that carry the bacteria represent a potential source of infection for people in close contact with them (129, 152) and might have a role in maintenance of the bacteria in the canine population (26).

Although bacteremia may be persistent for several months, fever is not a typical clinical feature in dogs naturally or experimentally infected. Infection with *B. canis* usually remains unnoticed when the dog does not have reproductive activity (35, 137, 182). Frequent findings in canine brucellosis during physical examination include long term enlargement of lymph nodes, especially the submandibular and retropharyngeal. The epididymis may be enlarged and firm, with scrotal dermatitis, and testicular atrophy. Orchitis has also been reported in *B. canis* infection, and although testicular swelling is infrequent and usually not detectable, pain on gentle palpation of testicles or epididymis may be noticed, and distention of tunica vaginalis cavity with fibrinopurulent exudate has been reported (35, 137, 182). Small testicular abscesses and prostate enlargement due to prostatitis with pelvic compression can be visualized by ultrasonography (137).

Loss of libido and male infertility has also been reported in *B. canis*-infected dogs (35). Semen from infected dogs may have sperm defects and head-to-head agglutination. Chronically infected dogs may be oligospermic or azoospermic (188). Auto antibodies against sperm contribute to infertility in infected dogs (189). However, the clinical manifestation may vary with less frequent clinical signs, especially in castrated dogs.

Congenitally infected puppies that survive or dogs infected later in life may present arthritis, ocular disease, discospondylitis, urinary retention (137), and osteomyelitis (190). Discospondylitis is the most common orthopedic disorder attributed to *B. canis* infection in dogs. In these cases, back pain, lameness, and neurologic deficits may be present. Radiography will show typical lesions and differential diagnosis with other infectious agents will require serology and/or isolation of *Brucella* from blood or lesions to address the appropriate treatment (184, 191–193).

Ocular lesions associated with *B. canis* infection have been reported and successfully treated in adult dogs. Ocular lesions were the exclusive complaint in three otherwise healthy dogs that presented recurrent blepharospasm and uveitis with hyperpigmentation of iridal surface, myosis, synechiae, lens capsule opacification and pigmentation, retinal lesions, vitreous opacity and optic disk hyperemia (183).

It has been reported by breeders that competing dogs presented loss of field-trial performance and poor coat quality after infection with *B. canis* (35).

## Laboratorial Diagnosis

This section discusses the most important diagnostic methods for canine brucellosis. A thorough review on diagnosis of human brucellosis has been recently published (194). As pointed out, a precise clinical diagnosis of canine brucellosis is not achievable. Therefore, laboratorial tests are essential for a definitive diagnosis. As in cases of other *Brucella* spp. infections, the gold standard for diagnosis of *B. canis* infection is isolation of the agent (19, 130) associated with biochemical tests (195–197) or more recently matrix-assisted laser desorption/ionization time-of-flight mass spectrometry (MALDI-TOF-MS) (198). *B. canis*, as well as other *Brucella* spp., grows well-under aerobic conditions on conventional media, such as dextrose or tryptic soy agar. However, considering its zoonotic potential, this procedure poses a considerable risk for laboratory personnel, requirinig biosafety level 3 conditions (151, 154, 199, 200).

In the absence of samples from aborted fetuses or vaginal secretions, whole blood is the sample of choice for *B. canis* isolation. In contrast to other *Brucella* spp., *B. canis* infection is associated with bacteremia that persists for 2–4 weeks, reaching up to 10^4^ CFU/mL of blood (19, 24, 130). Isolation of *B. canis* from blood samples may be done by direct or indirect culture methods (195). The use of liquid or biphasic media is recommended since *B. canis* may be found in very low numbers in blood samples (196). Importantly, regardless of the employed method, isolation of *B. canis* has low sensitivity, often resulting in false negative results. Some factors may further decrease the intrinsically low sensitivity of isolation, including: (i) antimicrobial treatment; (ii) use of EDTA, which inhibits bacterial growth (heparin or sodium citrate should be used as anticoagulant instead); and (iii) inadequate conditions for storage and transportation of samples (196, 201). Therefore, additional diagnostic methods are always recommended (195, 196).

In addition to blood, other samples are very useful for *B. canis* isolation. Vaginal and uterine secretions should be sampled during the proestrus and/or estrus, when there is an increased risk of bacteremia, or from bitches that have aborted (202). Samples of fetal membranes, aborted and stillbirth fetuses must be cultured when available, since these samples usually contain high bacterial loads. Semen samples should also be subjected to culture, particularly between 3 and 11 weeks after infection, when higher bacterial amounts are shed in the semen. After this period, shedding of *B. canis* in the semen becomes intermittent with low concentrations, and, therefore, cultures are often negative (18, 203). Urine samples are also useful for isolation, mostly between 8 and 30 weeks post infection. Concentrations of *B. canis* in the urine range from 10 to 10^2^ CFU/mL, and cystocentesis is the method of choice to prevent contamination (130, 202), although urine collected through the urethra may contain semen, which is an additional source of *B. canis* (202). In cases of *B. canis*-induced uveitis, aqueous humor is a suitable sample for culture, whereas in cases of discospondylitis or osteomyelitis, bone marrow aspirates are the samples of choice (65, 202, 204). At necropsy, several tissue samples should be sampled for culture, including lymph nodes, spleen, liver, and genital organs.

Direct diagnosis can also be achieved by detecting *B. canis* genomic DNA in biological samples by polymerase chain reaction (PCR) (205–208). This technique is faster than culture and it is not affected by bacterial viability or sample contamination (205, 209). Whole blood is the sample of choice for PCR, and although serum may also be used, it results in lower sensitivity (210). DNA extraction from blood samples must be performed with appropriate protocols to remove PCR inhibitors (208, 211–213). Importantly, the absence of bacteremia, antimicrobial drug usage, and PCR inhibitors (heparin) in blood samples, may also influence the result. Semen and tissue samples may also be employed for PCR (202).

PCR routinely used for diagnosis of canine brucellosis are genus-specific, targeting gene sequences that are conserved among *Brucella* spp. such as *bcsp31* (214) 16S ribosomal subunit (215, 216), and *recA* (217). PCR targeting these genes can be performed with DNA extracted from isolates or clinical samples. A species-specific PCR diagnosis can be achieved by multiplex PCR that requires purified bacterial DNA, and therefore are not applicable to clinical samples, requiring DNA extracted from isolates. Other techniques include the Bruce-Ladder PCR (218–220), the Suis-Ladder PCR (219), the HOOF-Prints PCR (221, 222), and the MLVA16 PCR (223–225). Real time quantitative PCR based on single nucleotide polymorphism (SNP) and high resolution melt (HRM) analysis on bcsp31 and 16S RNA ribosomal gene, allow identification of the genus *Brucella*, and in some cases the species, but this technique is currently restricted to the diagnosis of human brucellosis and for evaluation of treatment efficacy (226–232). Furthermore, these are expensive and labor intensive techniques.

Serologic tests are useful for diagnostic purposes since infected dogs remain serologically positive for several months even in the absence of bacteremia. Importantly, *B. canis* is serologically distinguished from *B. melitensis, B. abortus*, and *B. suis*, which carry a smooth LPS, and therefore their antigens do not react with anti-*B. canis* antibodies (195). However, none of the serological tests currently used for the diagnosis of canine brucellosis are completely satisfactory. Serologic diagnosis of *B. canis* infection is challenging, and a combination of different tests is highly recommended ideally in association with bacterial isolation (17, 202, 233). The serologic tests that are more frequently used for the diagnosis of *B. canis* infection include: rapid slide agglutination test (RSAT) (234), rapid slide agglutination test with 2-mercaptoethanol (2ME-RSAT) (235), and agar gel immunodiffusion test (AGID) (236). These tests detect antibodies against surface antigens of *Brucella* spp., particularly antibodies against rough LPS. Although these tests may have appropriate levels of sensitivity, false-positive results are common due to cross-reaction with other bacteria such as *Pseudomonas, Bordetella bronchiseptica, Streptococcus, Staphylococcus, Salmonella, Yersinia enterocolitica*, and *Escherichia coli* (19, 130, 231, 233, 237–239).

RSAT and 2ME-RSAT are serologic tests commonly used for screening of *B. canis* infection. The use of non-mucoid *B. canis* strains for antigen preparation may also decrease false-positive results (195, 202, 240). The tube agglutination test (TAT) is considered a semi-quantitative test and it is employed as a confirmatory test for RSAT or 2ME-RSAT (189, 202, 203), although false-positive or false-negative results are not uncommon (201, 233).

AGID, which is based on surface proteins as antigens, is capable of detecting precipitins between 5 and 10 weeks after infection. However, this method has important drawbacks including cross reactions and subjectivity for interpreting lines of precipitins (241). In addition to superficial antigens, cytoplasmic antigens may also be used for AGID, resulting in a highly specific test for *Brucella* spp. since cytoplasmic antigens are conserved only in organisms belonging to the genus *Brucella*. In this case, cytoplasmic antigens obtained by sonication of *B. canis* allow detection of antibodies in chronically infected dogs, even at 3 years post-infection in the absence of bacteremia. However, in acute infections, cytoplasmic antigen-based AGID tends to detect precipitins at later stages of infection when compared to surface antigen-based AGID (201, 203, 204).

Several enzyme-linked immunosorbent assay (ELISA) protocols have been applied to the diagnosis of *B. canis* infection, but sensitivity and specificity varies according to the antigen used (204, 238, 241–244). Antigens employed in ELISA protocols include: *B. canis* surface antigens (245), cytoplasmic antigens (242), antigens extracted by heated saline solution (HSS) from non-mucoid *B. canis* (M-variant), etc (238). Indirect ELISA is considered more specific, but less sensitive than TAT for screening (203). However, this method is more sensitive than agglutination methods and AGID (19, 204, 246). Furthermore, ELISAs can detect antibodies in chronically infected dogs that that test negative by 2ME-RSAT and AGID (247). ELISA can detect antibodies at 30 days post infection, and it may be useful as a confirmatory test (93, 98, 241).

The complement fixation test (CFT) is considered a confirmatory test for *B. ovis* and *B. abortus* infection (248–251). Although it has high specificity and sensitivity, CFT has not been routinely used for diagnosis of canine brucellosis (252). Immunochromatographic assays have been developed for the diagnosis of *B. canis* infections (247, 253, 254). These are simple and rapid assays, but they have low sensitivity when compared to other traditional screening methods (247).

Regardless of the serologic method employed, false-negative results are commonly observed during the first 3 to 4 weeks after infection, even when bacteremia is present. Therefore, dogs should be tested at least twice in 30 days intervals. Considering different serologic methods, dogs remain positive for 8–12 weeks after infection (202). Serum samples should be preferably obtained from bitches at proestrus, estrus or during gestation or immediately after abortion (202). Serum samples must be free of hemolysis since hemoglobin may result in agglutination and, consequently, false-positive results (235).

## Treatment

### Treatment in Humans

The treatment of brucellosis in humans is based on the use of antibiotics capable to act in intracellular medium for an adequate length of time (156), including doxycycline (138, 153, 157, 159, 163–165, 255), streptomycin (143, 150, 153, 154, 256, 257), rifampicin (129, 138, 157, 159, 163, 255), gentamicin (138, 160, 165), trimethropim-sulfametoxazole (129, 138, 150, 152), ofloxacin (157), ciprofloxacin (164), tetracycline (23, 255, 258), ampicillin (138, 143, 160, 259), sulfadiazine (154), ceftriaxone (152) and cephalothin (138, 160).

In the first reports of human infection with *B. canis*, Morisset and Spink (23) and Munford et al. (259) described the use of monotherapy with tetracycline and ampicillin, respectively. However, the association of two or more antibiotics is considered the most consistent and effective treatment due to the high relapse rates of monotherapy (260). The treatment is usually prolonged and varies (up to 6 weeks) according to antibiotics (156).

The treatment for children with doxycycline and tetracycline is not recommended due to the irreversible staining of the teeth (9, 260) and inhibition of bone growth (9). Cotrimoxazole and rifampicin are not indicated for use in young children, and the use of these drugs separately in monotreatment commonly results in treatment failure (9). In this case, the treatment is usually based on the association of trimethoprim-sulfamethoxazole with rifampicin for 4–6 weeks, with no negative effects on the efficacy of treatment (129).

In pregnant woman, tetracyclines are contraindicated due to permanent staining of fetal dentition and the potential to induce necrosis of the liver and pancreatitis (9). Sulfamethoxazole and trimethoprim individually or combined (cotrimoxazole) should be avoided during gestation. These drugs are potentially neurotoxic for the fetus due to the elevation of plasma bilirubin that reaches the central nervous system causing kernicterus (256). In some cases of complication due to *Brucella* spp. infection as osteoarticular impairment and endocarditis, the treatment needs to be prolonged, and relapses in these cases are common (9, 157, 257).

### Treatment in Dogs

In dogs, the treatment with antibiotics is not encouraged, especially due to the high rates of relapse, and the cure for the disease still uncertain after antibiotic treatment, resulting in high risk of transmission to humans and other dogs (16, 261). In addition, expensive antibiotic may be prohibitive for some owners (262). It is important to highlight that antibiotic therapy does not completely eliminate *B. canis*. Therefore, absence of the clinical signs after treatment does not indicate the absence of the bacterium (263).

*B. canis* isolated from dogs are usually susceptible to doxycycline and tetracycline (264, 265), whereas some *B. canis* strains are considered more resistant to streptomycin and tetracycline than other *Brucella* spp. (264). Importantly, enrofloxacin and streptomycin have synergic activity *in vitro* against the bacteria, while doxycycline and rifampicin have antagonistic effects (265).

Treatment with oxytetracycline for 4 weeks and streptomycin in the 1st week of treatment results in effective treatment in 79% of dogs, when elimination of bacteremia and absence of *B. canis* in lymph nodes, spleen and reproductive organs are considered (262). Enrofloxacin have good results to prevent occurrence of abortion, with results that are similar to streptomycin, which may be toxic and is not indicated during pregnancy (263).

## Control and Prevention

*B. canis* infection causes infertility in dogs (24, 137) and has progressively gained more attention from dog breeders due to significant economic losses as well as the public health risk (24, 38).

Unfortunately, there is not any commercially available vaccine for prevention of canine brucellosis. Therefore, control measures include (i) screening tests for dogs and kennels suspected of having brucellosis, (ii) treatment or euthanasia of infected dogs, and (iii) elimination of the bacteria from the environment (24, 137).

In commercial kennels, dogs should be subjected to screening tests annually or twice a year, and in case of positive or inconclusive results, dogs must be subjected to quarantine and confirmatory tests (24, 202). In case of confirmation of the diagnosis, euthanasia should be considered (24).

Precaution is required when introducing new dogs into a kennel. Newly acquired dogs must remain isolated for at least 1 month, and they should only be introduced in the kennel after two negative diagnostic test results with an interval of one month (24, 202, 204, 245, 266–268). Dogs with clinical signs compatible with brucellosis should not be acquired (202). Besides, dogs from a positive kennel should be monthly tested for at least 3 months after becoming negative, particularly prior to breeding (24, 137, 202). Importantly, since canine brucellosis is a zoonosis with high occupational risk, owners and/or kennel employees must be properly educated and protected, mostly in order to prevent contact with infected dogs and secretions, especially during parturition or abortion. Personnel must be aware of *B. canis* infection in the kennel and be subjected to diagnostic tests or treatment if needed (137, 202).

Elimination of *B. canis* from the environment is another very important control procedure. *B. canis* does not survive for prolonged periods under environmental conditions. It is quickly killed by most disinfectants including 1% sodium hypochlorite, 70% ethanol, ethanol/iodine solutions, glutaraldehyde, and formaldehyde (24, 137). However, organic matter and low temperatures may impair disinfectant efficiency. Surfaces may be decontaminated with 2.5% sodium hypochlorite, which should be maintained over the surface for at least 1 h. Decontamination of body surfaces may be done with 70% ethanol or iodine solutions. Equipment may be decontaminated by autoclaving at 121°C for at least 15 min or by dry heat at 160–170°C for at least 1 h. Boiling for 10 min also inactivates *Brucella* (269).

In case of pet dogs infected with *Brucella*, owners must be informed about the zoonotic risks before choosing treatment or euthanasia. Orchiectomy or ovary-hysterectomy should be considered to eliminate the primary site of infection and decrease the risk of transmission. In addition, infected dogs must be treated since the pathogen remains in tissues of castrated dogs in spite of a lower risk of transmission (24, 202). Electing treatment instead of euthanasia should be performed only under rigorous supervision of a veterinarian, and in case of treatment is chosen, infants should not be in contact with the infected dog (270). Dog owners must also be aware that even under stringent conditions, treatment may not result in cure, and a second round of treatment may be required (24, 202). Treated dogs must be tested again, and negative results by serology, PCR and bacterial isolation may be interpreted as a presumptive of cure (202). In addition, measures for controlling environmental contamination should be applied to households (24, 137).

Good practices for controlling zoonotic diseases in general play an important role in controlling canine brucellosis. Therefore, contraceptive methods, preferably surgical sterilization, for stray dog populations are important in this context.

In spite of a significant research effort in the field of *Brucella* vaccinology (271), studies specifically aiming the development of a vaccine for canine brucellosis are scarce and restricted to the last decade (272, 273). Strategies employed for experimental vaccines include inactivated vaccines (273), a recombinant outer membrane proteins (Omp31) (274, 275), recombinant VirB proteins (276), attenuated mutant vaccine strains such as a *B. canis* mutant in SST4 (272) and a mutant versions of *B. abortus* RB51 vaccine strain (277). However, despite its attenuation, the vaccine strain *B. abortus* RB51 retains pathogenic potential (277). Live attenuated vaccines provide the highest levels of protection (271). Indeed, recently two new vaccine candidates for canine brucellosis have emerged, namely a mutant strain *B. canis* Δ*vjbR* (278) and *B. ovis* Δ*abcBA* (279). This later vaccine strain has been developed by our group taking advantage of the antigenic similarities between *B. canis* and *B ovis*, utilizing a background that has no zoonotic potential since there is not a reported case of human brucellosis attributed to *B. ovis*, and no residual pathogenicity for animals including sheep (280). A recent study demonstrated that the vaccine candidate *B. ovis* Δ*abcBA* protects against experimental challenge with *B. canis* in mice, and when this vaccine strain is encapsulated in alginate and administered to dogs, it is not shed in the semen or urine and is safe (279).

## Future Perspectives

Diagnosis of *B. canis* infection is very challenging. Although the dog is the most common host of *B. canis*, canine infections with other *Brucella* spp. such as *B. suis* (281) and *B. abortus* (282) may occur. Therefore, the development of effective and accurate *B. canis*-specific diagnostic methods is extremely relevant.

Currently, prevention and control of canine brucellosis are not easily achieved, especially due to the difficulty in identifying infected dogs, which could prevent spreading of the disease. In this context, development of novel diagnostic methods is highly desirable as well as the development of efficacious and safe vaccines.

In addition to the development of novel diagnostic methods and vaccines for the control of canine brucellosis, which will be a turning point in controlling this disease, raising awareness among human health professionals could have a significant impact. This may lead to a better knowledge of the impact of human brucellosis associated with *B. canis*, allowing proper therapeutic interventions and mitigation of deleterious effects of the disease.

## Author Contributions

RS and TP contributed revising the manuscript and acting as senior authors. All authors contributed to conceptualize, write the manuscript, contributed writting the body of the manuscript, and approved the manuscript for publication.

## Conflict of Interest

The authors declare that the research was conducted in the absence of any commercial or financial relationships that could be construed as a potential conflict of interest.
