# Supplementary material for: Canine Brucellosis: An Update
Source: Front Vet Sci. 2021 Mar 2;8:594291. doi: 10.3389/fvets.2021.594291 (PMC7962550; doi:10.3389/fvets.2021.594291)
Supplement: Supplementary file 1 [file Table_1.pdf]

Supplementary Material

Supplementary Table 1. Epidemiologic surveys and frequency of canine brucellosis in dogs around the world.

Country	Year	Sampling based in clinical signs presentation?	Positive Frequency	Diagnostic Method	Reference
Argentina	NI[1]	No	14.70% (33/224)	RSAT[2]	López et al. (2009)[40]
Argentina	NI	No (Stray dogs)	30.50% (40/131)	Tube agglutination test	Myers and Varela-Díaz (1980)[41]
Argentina	NI	No	7.30% (16/219)	AGID[4] (*B. ovis* as antigen)	Boeri et al. (2008)[42]
Australia	1985	No	5.50% (18/325)	RSAT	Medveczky and Crichton (1986)[43]
Austria	2010	Yes (Iritis, Fouetal dead with history of retarded growth and no more heart activity)	NA[3]	Tube agglutination test /Bacterial isolation	Hofer et al. (2012) [28]
Austria	2011–2016	No (random sampling)	11.6% (11/95)	2ME Tube agglutination test	Buhmann et al. (2019) [44]
Bangladesh	2009	No	6.67% (2/30)	Tube agglutination test	Talukder et al. (2011) [45]
Belgium	2011–2016	No (random sampling)	12.20% (6/49)	2ME Tube agglutination test	Buhmann et al. (2019) [44]
Brazil	2014-2016	Puppies of breeding kennels	21% (42/200)	PCR	Souza et al. (2018) [46]

Brazil	2001	No (random sampling)	14.2% (90/635)	AGID (*B. ovis* as antigen)	Almeida et al. (2004) [47]
Brazil	NI	No (random sampling)	1.3% (1/76)	Tube agglutination test	Godoy et al. (1977) [48]
Brazil	1999-2000	No (random sampling)	4.8% (65/1368)	AGID (*B. ovis* as antigen)	Souza et al. (2002) [49]
Brazil	2008-2010	No (random sampling)	0.0% (0/140)	AGID (*B. ovis* as antigen)	Castro et al. (2014) [50]
Brazil	1998	No (random sampling)	0.84% (19/1072)	2ME RSAT	Moraes et al. (2002a) [51]
Brazil	2007-2008	No (random sampling)	1.05% (6/570)	Bacterial Isolation	Mascolli et al. (2016) [52]
Brazil	2003-2004	No (random sampling)	0.8% (4/500)	AGID (*B. ovis* as antigen)	Reis et al. (2008) [53]
Brazil	NI	No (random sampling)	3.6% (8/221)	Tube agglutination test	Sandoval et al. (1976)[54]
Brazil	NI	NI	7.0% (14/200)	Tube agglutination test	Larsson et al. (1981) [55]
Brazil	1981-1985	Cross sectional study	7.5% (254/3386)	AGID (antigen non identified)	Cortes et al. (1988) [56]
Brazil	2000-2002	No (dogs from comercial kennels)	33.9% (58/171)	AGID (*B. ovis* as antigen)	Keid et al. (2004) [57]
			14.0% (24/171)	Bacterial Isolation	
Brazil	2003-2007	Diagnosis requested by owners of dogs	20.9% (158/753)	Bacterial Isolation	Keid et al. (2017) [58]
Brazil	1999	No (random sampling)	2.2% (9/410)	2ME AGID (*B. ovis* as antigen)	Azevedo et al. (2003) [59]

Brazil	NI	No (random sampling)	3.3% (3/90)	2ME AGID (*B. ovis* as antigen)	Porto et al. (2008) [60]
Brazil	NI	No (random sampling)	5.8% (5/85)	AGID (*B. ovis* as antigen)	Cavalcanti et al. (2006) [61]
Brazil	NI	No (random sampling)	33.8% (60/177)	iELISA	Oliveira (2008) [62]
Brazil	NI	No (random sampling)	37.0% (40/108)	AGID (*B. ovis* as antigen)	Melo et al. (1997) [63]
Brazil	2003	No (random sampling)	2.3% (4/170)	AGID (*B. ovis* as antigen)	Vasconcelos et al. (2008) [64]
Brazil	2008-2009	No (random sampling)	3.1% (6/193)	AGID (*B. ovis* as antigen)	Fernandes et al. (2011) [65]
Brazil	NI	No (random sampling)	7.4% (44/591)	AGID (*B. ovis* as antigen)	Lima et al. (2014) [66]
Brazil	2011	No (random sampling)	28.8% (120/416)	AGID	Fernandes et al. (2013) [67]
Brazil	2008	No (random sampling)	1.6% (1/60)	AGID (*B. ovis* as antigen)	Lima (2009) [68]
Brazil	2013	No (random sampling)	0.0% (0/311)	2ME AGID (*B. ovis* as antigen)	Paz et al. (2015) [69]
Brazil	NI	No (random sampling)	0.0% (0/304)	2ME AGID (*B. ovis* as antigen)	Aguiar et al. (2005) [70]
Brazil	2005	No (random sampling)	44.6% (167/374)	AGID (*B. ovis* as antigen)	Dorneles et al. (2011) [71]
Brazil	2008	No (random sampling)	54.7% (132/241)	AGID (*B. ovis* as antigen)	Santana et al. (2013) [72]
Brazil	2007-2008	No (random sampling)	24.1% (79/327)	PCR	Silva et al. (2012a) [73]

Brazil	2017-2018	No (random sampling)	4.34% (2/46)	AGID (*B. ovis* as antigen)	Petry et al. (2019)[74]
Brazil	NI	No (random sampling in shelter dogs)	4.0% (4/100)	AGID (*B. ovis* as antigen)	Silva et al. (2012b) [75]
Brazil	2013	Yes (dogs with reproductive problems)	5.4% (1/22)	AGID (*B. ovis* as antigen)	Machado (2013) [76]
Brazil	2011	No (Cross-sectional study)	2.8% (5/175)	AGID (*B. ovis* as antigen)	Dreer et al. (2013) [32]
Brazil	NI	No (random sampling)	11.9% (23/192)	Tube agglutination test	Wald and Fernandes (1976) [77]
Canada	NI	No	0.30% (100/2000)	RSAT	Bosu and Prescott. (1980) [78]
Canada	1979	No (random sampling)	1.60% (6/341)	RSAT	Higgins et al. (1979) [79]
Chile	2008	Pets from immunocompromised children	2.43% (1/41)	ELISA	Abarca et al. (2011) [80]
Chile	2011	No (Cross-sectional study)	1.00% (4/400)	Immunochromatography (commercial kit Brucella IC® Biopronix-Italia)	Tuemmers et al. (2013) [81]
Chile	1978	No (random sampling)	21.84% (38/174)	Tube agglutination test	Zamora et al. (1980) [82]
Chile	2013-2014	Pets from immunocompromised children	14.28% (5/35)	Commercial Immunofluorescence assay (Fuller Laboratories, Fullerton, CA, USA)	Peña et al. (2016) [83]
China	2011	Yes, outbreak of abortion in a breading farm	48.75 (39/80)	RSAT	Jiang et al. (2011) [84]

Colombia	NI	No (random sampling)	15.3% (59/385)	2ME RSAT	Sánches-Jimenez et al. (2020) [85]
Colombia	2011	No (Cross-sectional study in kennels)	14.90% (64/428)	RSAT	Castrillón-Salazar et al. (2013) [86]
Colombia	2009	No (Cross-sectional study)	2.76% (12/441)	Immunochromatography (Antigen Rapid C Brucella Ab Test Kit)	Agueldo-Florez et al. (2012) [87]
Colombia	NI	No (Cross-sectional study)	1.49% (3/201)	RSAT	Pardo et al., (2009) [88]
Denmark	2011–2016	No (random sampling)	5.10% (6/117)	2ME Tube agglutination test	Buhmann et al. (2019) [44]
Finnish	2002-2004	Voluntieer dogs	0 (0/388)	Tube agglutination test and RSAT	Dahlbom et al. (2009) [89]
Finland	2011–2016	No (random sampling)	6.9% (14/203)	2ME Tube agglutination test	Buhmann et al. (2019) [44]
France	2011–2016	No (random sampling)	0.8% (3/382)	PCR	Buhmann et al. (2019) [44]
			2.7% (11/415)	2ME Tube agglutination test	
Germany	NI	No (random sampling)	0.2% (2/1000)	Tube agglutination test	Weber and Schliesser (1978) [90]
Germany	NI	Abortion	50.0% (100/200)	Clinical diagnosis	Von Kruedener (1976) [91]
Germany	NI	No	0.6% (5/830)	Tube agglutination test	Wintermantel (1980) [92]
Germany	2011–2016	No (random sampling)	0.0% (0/386)	PCR	Buhmann et al. (2019) [44]
			5.4% (58/1065)	2ME Tube agglutination test	
Hungary	2011–2016	No (random sampling)	5.10% (6/140)	2ME Tube agglutination test	Buhmann et al. (2019) [44]
India	NI	No (random sampling)	2.27% (12/527)	2ME Tube agglutination test	Barkha et al. (2011)

			1.5% (8/527)	AGID (*B. canis* as antigen)	[93]
			3.03% (16/527)	dot-ELISA	
India	NI	No (random sampling)	2.25% (9/400)	Tube agglutination test	Lingam et al. (2020) [94]
Iran	2006-2008	No (random sampling)	4.90% (5/102)	Immunochromatography (C. *Brucella* Ab test kit; Animal Genetics, Korea)	Mosallanejad et al. (2009) [95]
Iran	NI	No (random sampling)	10.62% (12/113)	Immunochromatography (C. *Brucella* Ab test kit; Animal Genetics, Korea)	Behzadi and Mogheiseh (2011) [96]
Iran	2009-2010	No (random sampling)	15.8% (15/95)	Commercial Immunofluorescence Kit MegaSreen FLUOBRUCELLA (MegaCor, Horbranz, AT)	Akhtardanesh et al. (2011) [97]
Italy	1994-2001	Dogs showed clinical signs but not specified by the authors	1.07% (25/2328)	AGID (*B. canis* antigen)	Ebani et al. (2003) [98]
Italy	2011–2016	No (random sampling)	1.0% (1/103)	PCR	Buhmann et al. (2019) [44]
			7.9% (17/215)	2ME Tube agglutination test	
Japan	2009-2017	No (random sampling)	3.5% (38/1071)	RSAT	Nabeshima et al. (2020) [99]
Japan	1974-1977	No (random sampling)	2.90% (27/945)	RSAT	Saegusa et al. (1978) [100]
Japan	1974	No (random sampling)	0.92% (5/540)	RSAT using heat-killed *B. canis* as antigen	Takayoshi et al. (1977) [101]
Japan	1989-1990	No (random sampling)	1.90% (5/259)	RSAT using heat-killed *B. canis* as antigen	Katami et al. (1991) [102]
Japan	1976-	No (random sampling)	11.20%	RSAT	Kikuchi et al. (1979)

	1977		(173/1549)		[103]
Japan	2003-2006	No (random sampling)	2.50% (12/485)	Micro agglutination test	Kimura et al. (2008) [104]
Jordan	NI	No (random sampling)	8.3%(14/169)	2ME RSAT	Alshehabat et al. (2019) [105]
Mexico	NI	No (random sampling)	28.00% (140/500)	RSAT and Tube agglutination test	Flores-Castro e Segura (1976) [29]
			1.6% (8/500)	Bacterial isolation	
Mexico	1990-1991	No (random sampling)	0.50% (1/200)	RSAT	Lara-Lara et al. (1993) [106]
Mexico	NI	Yes, there was considered dogs that present one or more clinical signs including: epididymitis, scrotal dermatitis, unilateral or bilateral testicular atrophy, infertility, lymphadenitis, diskospondylitis and/or uveitis	42.80% (24/56)	RSAT	González et al. (2004) [107]
Mongolia	2010	Cross-sectional study	36.40% (26/72)	RSAT	Zolzaya et al. (2014) [108]
Netherlands	2011–2016	No (random sampling)	2.0% (2/98)	2-ME Tube agglutination test	Buhmann et al. (2019) [44]
New Guinea	1984-1986	No (random sampling)	0.40% (1/225)	Tube agglutination test	Patten (1987) [19]
New Zealand	1992-	Clinal signs NI	0% (0/90)	Bacterial isolation	Gardner and Reichel

	1993	No (random sampling)	0% (0/500)	ELISA	(1997) [37]
Nigeria	1982-1984	No (random sampling)	28.60% (64/224)	2-ME Tube agglutination test	Adesiyun et al. (1986) [110]
Nigeria	NI	No (random sampling)	0.27% (1/366)	RSAT	Cadmus et al. (2011) [111]
Nigeria	NI	No (random sampling)	27.64% (34/123)	Dot-ELISA (commertial kit Biogal-Galed Laboratories, Israel)	Anyaoha et al. (2020) [112]
Norway	2011–2016	No (random sampling)	4.10% (3/73)	2-ME Tube agglutination test	Buhmann et al. (2019) [44]
Pakistan	2015-2016	No (conveniente sampling)	37.6 (68/181)	Tube agglutination test	Jamil et al. (2019) [113]
Poland	2011–2016	No (random sampling)	6.7% (29/432)	PCR	Buhmann et al. (2019) [44]
			3.7% (6/164)	2-ME Tube agglutination test	
South Africa	2005	No (Cross-sectional study)	9.75% (39/400)	Tube agglutination test	Etsebeth (2017) [114]
South Korea	2008	No (random sampling)	2.5% (10/402)	Immunochromatography (C. *Brucella* Ab test kit; Bionote co., Korea)	Hong et al. (2009) [115]
South Korea	2015-2016	No (random sampling)	1% (30/2394)	Immunochromatography (C. *Brucella* Ab test kit; Bionote co., Korea)	Jung et al. (2018) [116]
Spain	NI	No (random sampling)	20.20% (70/346)	RSAT (*B. ovis* antigen)	Mateu-de Antonio et al. (1994) [117]
			6.1% (21/346)	2ME RSAT (*B. ovis* antigen)	
			8.7% (30/346)	RSAT (*B. canis* as antigen)	
			5.8% (20/346)	2ME RSAT (*B. canis* as antigen)	

			7.8% (27/346)	2ME Tube agglutination test	
			14.5% (50/346)	AGID (*B. canis* as antigen)	
Spain	2011–2016	No (random sampling)	11.10% (28/253)	PCR	Buhman et al. (2019) [44]
Sweden	2013	Yes (outbreak of abortion in kennel)	12% (3/25)	qPCR	Kaden et al. (2014) [118]
Sweden	2011–2016	No (random sampling)	13.6% (3/22)	2-ME Tube agglutination test	Buhmann et al. (2019) [44]
Switzerland	2017	NA	NA	RSAT/Bacterial isolation	Egloff et al. (2018) [119]
Switzerland	2011–2016	No (random sampling)	8.20% (7/85)	2ME Tube agglutination test	Buhmann et al. (2019) [44]
Turkey	2007-2008	No (dogs that died were studied)	8.30% (4/48)	PCR/ Bacterial isolation	Aras and Uçan (2010) [120]
Turkey	NI	No (random sampling)	12.70% (46/362)	Tube agglutination test	Onçel et al. (2005) [121]
			7.73% (28/362)	Tube agglutination test -2ME	
			7.45% (27/362)	ELISA (Sonicated *B. canis* as antigen)	
United Kingdom	NI	No (random sampling)	28.40% (48/169)	RSAT	Taylor (1980) [122]
United States	1973-1974	No (random sampling)	9.40% (22/235)	Tube agglutination test	Lovejoy et al. (1976) [30]
United States	1995-2005	Yes, outbreaks in 4 kennels	1.96%/ (10/510)	RSAT	Brower et al. (2007) [27]
			4.60% (8/174)	Pulsed-field gel electrophoresis	
United States	NI	No (random sampling)	0.50% (10/200)	RSAT	Brown et al. (1976) [31]

United States	NI	No (random sampling)	6.70% (17/2572)	RSAT	Boebel et al. (1979) [123]
			1.5% (41/2572)	Tube agglutination test	
			0.2% (6/2572)	Bacterial isolation	
United States	NI	Dogs submitted to eutanasia	3.65% (10/274)	Tube agglutination test	Hoff et al. (1974) [124]
Zimbabwe	NI	Cross-sectional study	17.60% (57/324)	Dot-ELISA (commertial kit Biogal-Galed Laboratories, Israel)	Chinyoka et al. (2014) [125]

NI[1]: Non informed. RSAT[2]: Rapid slide agglutination test. NA[3]: not applicable (case reports without epidemiological survey). AGID[4]: Agar gel immunodifusion
